# *para*-Hydrogen-Induced Polarization Enabled by Visible Light Activation

**DOI:** 10.1021/acscentsci.2c01341

**Published:** 2022-11-30

**Authors:** Su Chen, Shengtong Niu, Wei Liu

**Affiliations:** Department of Chemistry, University of Cincinnati, Cincinnati, Ohio 45221, United States

Nuclear magnetic resonance (NMR) spectroscopy is an effective technology for chemical analysis and medical diagnosis. However, a significant weakness of NMR is its limited sensitivity owing to the low spin polarization. In fact, only a few parts per million of all spins in a sample can contribute to the NMR signals. In a recent issue of *ACS Central Science*,^1^ Castellano, Theis, and co-workers reported a visible-light-induced hyperpolarization approach that can enhance the signal of ^1^H NMR spectroscopy by 3 orders of magnitude. Traditional approaches to increase the NMR signal involve the use of high magnetic fields, long acquisition times, or cryogenically cooled detectors.^2^ A new form of NMR has emerged recently based on the concept of hyperpolarization, in which the nuclear spins are polarized to a level far beyond the thermal equilibrium.^3^ Hyperpolarization can improve nuclear spin polarization by ∼4–5 orders of magnitude.^4^ Among the many hyperpolarization techniques, *para*-hydrogen-induced polarization (PHIP)^5^ is a scalable and fast method that relies on the pairwise addition of *para*-hydrogen (*p*-H_2_)—a singlet spin isomer of H_2_—across unsaturated chemical bonds.^6,7^ To date, catalytic hydrogenation under thermal conditions represents the most frequently applied method for the installation of *para*-hydrogen.^8^ A few examples have been reported that could enable PHIP via light activation, but the use of deep ultraviolet light imposes restrictions on further applications.^9^

**Figure 1:**
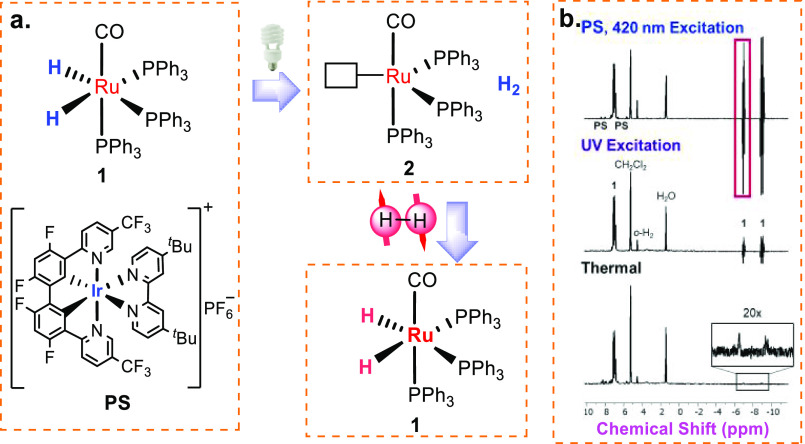
(a) Ru(CO)(PPh_3_)_3_(H)_2_**1** can be activated photochemically by an Ir(III) triplet photosensitizer (PS) under visible-light excitation. A Ru(0) complex **2** is formed as the intermediate in the photoactivation reaction. (b) ^1^H NMR signal enhancement of **1**. Reproduced with permission from ref (1). Copyright 2022 American Chemical Society.

Castellano, Theis, and co-workers questioned whether PHIP could be induced by visible light, thus making the hyperpolarization process occur in a controllable manner. In this proof-of-concept work, the authors have ingeniously demonstrated that this goal could be achieved by using a Ru(II) dihydride complex, Ru(CO)(PPh_3_)_3_(H)_2_, as the hydrogenation catalyst. This Ru(II) complex is a d^6^-based octahedral transition metal complex with a triplet ligand-field state and can be activated photochemically (Figure 1). At room temperature, the excitation of **1** with either visible light (420 or 427 nm) in the presence of an Ir(III) triplet photosensitizer (PS) or UV light (365 nm) alone resulted in the loss of both hydrides and the formation of a Ru(0) intermediate **2**. Under a *p*-H_2_ atmosphere (3 atm), this Ru(0) intermediate could undergo oxidative addition to reform the dihydride complex **1** bound with two *p*-H_2_-derived hydride ligands (Figure 1a). Visible light excitation of 1 led to a 235-fold enhancement of the ^1^H NMR signals for the hydride atoms. In comparison, direct excitation of **1** at 365 nm in the absence of the PS yielded a 35.6-fold enhancement of the same signals (Figure 1b).

Subsequently, the catalytic performance of **1** for hydrogenation was carefully studied. Transient absorption spectroscopy revealed that the transiently produced Ru(0) species could bind with phenylacetylene with a second order rate constant of *k* = 7.47 ± 0.12 × 10^5^ M^–1^ s^–1^. The coordination of phenylacetylene to the Ru(0) intermediate, along with the photoactivable nature of **1**, encouraged authors to investigate the possible hydrogenation reaction with **1** as the catalyst. In a model reaction, the authors have demonstrated that **1** could catalyze the hydrogenation of phenylacetylene at room temperature under UV light activation. Styrene was formed as the main hydrogenation product, with a turnover number of 4.16 within 1 h. Other unsaturated substrates, including *p*-tolylacetylene and ethylpropiolate, could also undergo hydrogenation under the photocatalyzed conditions. Despite the fair catalytic performance, these results clearly revealed that Ru(CO)(PPh_3_)_3_(H)_2_ catalyzes the hydrogenation of unsaturated organic molecules under triplet photosensitized conditions at room temperature.

**Figure 2:**
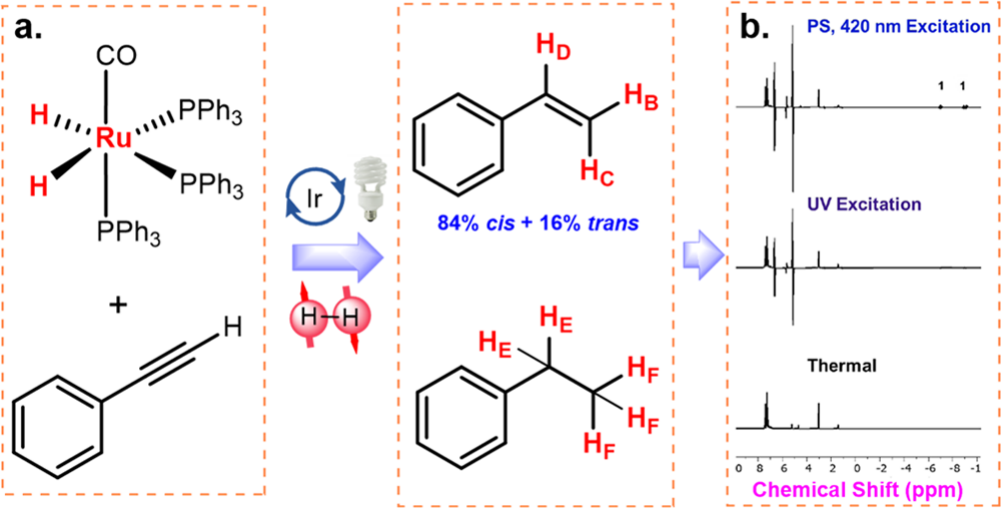
(a) Process of **1** hyperpolarizes phenylacetylene in the presence of 3 atom *p*-H_2_ with visible-light irradiation. (b) ^1^H NMR spectra for the hydrogen atom in styrene from *p*-H_2_. Reproduced with permission from ref (1). Copyright 2022 American Chemical Society.

After demonstrating the photoactivated catalytic performance of **1**, the authors took a step forward and examined the effectiveness of **1** for the transfer of hyperpolarization to organic molecules. To this end, the catalytic hydrogenation of phenylacetylene was conducted in the presence of *p*-H_2_ (3 atm). Under either visible light activation with the PS or direct UV excitation, hydrogenation of phenylacetylene catalyzed by **1** led to the efficient hyperpolarization of the hydrogenated products at room temperature. A 1630-fold enhancement in the ^1^H NMR signals for the hydrogen atom in styrene was observed with respect to the thermally equilibrated samples, whereas direct excitation of the same reaction mixture with UV light yielded a 906-fold increase in the signals (Figure 2). What is more intriguing is that the transfer of hyperpolarization could be achieved at even lower temperatures (0 °C), resulting in a 226-fold increase of ^1^H NMR signals compared with the thermally equilibrated samples.

This work by Castellano, Theis, and co-workers constitutes the first example of visible-light-enabled PHIP and represents a milestone in the fields of both photochemistry and hyperpolarization. In comparison with the traditional methods that rely on either thermal approaches or deep UV light activation, the use of visible light allows for the generation of hyperpolarization with higher efficiency and selectivity. Indeed, this photosensitized PHIP allows hyperpolarization within only 0.8 s of visible light excitation. The enhancement of NMR signals under mild conditions could also help to investigate reaction mechanisms, shed light on homogeneous and heterogeneous catalysis, and be used to study various metabolic pathways. Furthermore, given the wide application of hyperpolarization techniques in magnetic resonance imaging (MRI), the new approach reported here could also open a research area for the development of light-activated MRI contrast agents.
